# Artificial Intelligence in Oncologic Thoracic Surgery: Clinical Decision Support and Emerging Applications

**DOI:** 10.3390/cancers18020246

**Published:** 2026-01-13

**Authors:** Francesco Petrella, Stefania Rizzo

**Affiliations:** 1 Department of Thoracic Surgery, Fondazione IRCCS San Gerardo dei Tintori, 20900 Monza, Italy; francesco.petrella@irccs-sangerardo.it; 2 Department of Oncology and Hemato-Oncology, University of Milan, Via Festa del Perdono 7, 20122 Milano, Italy; 3 Clinic of Radiology EOC, Via Tesserete 46, 6900 Lugano, Switzerland; 4 Faculty of Biomedical Sciences, Università della Svizzera Italiana (USI), Via G. Buffi 13, 6900 Lugano, Switzerland

**Keywords:** artificial intelligence, thoracic surgery, lung cancer, diagnosis, patient outcomes

## Abstract

Artificial intelligence (AI) is rapidly reshaping thoracic surgery, advancing from decision support to the threshold of autonomous intervention. AI-driven technologies—including machine learning (ML), deep learning (DL), and computer vision—have demonstrated significant improvements in diagnostic accuracy, surgical planning, intraoperative navigation, and postoperative outcome prediction. In lung cancer and thoracic oncology, AI enhances imaging analysis, histopathological classification, and risk stratification, supporting multidisciplinary decision-making and personalized therapy. Robotic-assisted and AI-guided systems are optimizing surgical precision and workflow efficiency, while real-time decision-support tools and augmented reality are improving intraoperative safety. Despite these advances, widespread adoption is limited by challenges in algorithmic bias, data integration, regulatory approval, and ethical transparency. The literature emphasizes the need for multicenter validation, explainable AI, and robust governance frameworks to ensure safe and effective clinical integration. Future research should focus on digital twin technology, federated learning, and transparent AI outputs to further enhance reliability and accessibility. AI is poised to transform thoracic surgery, but responsible implementation and ongoing evaluation are essential for realizing its full potential. The aim of this review is to evaluate and synthesize the current landscape of artificial intelligence (AI) applications across the thoracic surgical pathway, from preoperative decision-support to intraoperative guidance and emerging autonomous interventions.

## Simple Summary

Artificial intelligence (AI) is changing the way surgeons perform chest surgery, including operations on the lungs and other organs in the chest. AI uses computers to help surgeons make better decisions, plan surgeries, and even guide them during operations. For example, AI can help spot lung cancer earlier by analyzing scans and test results more quickly and accurately than before. During surgery, smart robots and computer programs can help surgeons be more precise, making the surgery safer and helping patients recover faster. After surgery, AI can help physicians watch for problems and predict which patients might need extra care, using information from wearable devices and medical records. While these new tools are promising, there are still challenges. AI needs to be tested widely to make sure it works for everyone, and physicians must be careful about privacy and fairness. Some computer programs can make mistakes or be hard to understand, so experts are working to make AI safer and easier to use. Overall, AI is helping medical doctors to improve care for people who need chest surgery, but more research and careful planning are needed before these tools become a regular part of medical practice.

## 1. Introduction

Artificial intelligence (AI) is reshaping the landscape of thoracic surgery, offering transformative capabilities across the entire surgical continuum. Recent advances in machine learning, deep learning, and computer vision have enabled AI-driven systems to enhance diagnostic accuracy, optimize preoperative planning, and support intraoperative decision-making, particularly in complex procedures such as lung cancer resection and mediastinal interventions [1,2,3]. AI-powered imaging analysis and radiomics have improved detection and classification of pulmonary nodules, prediction of lymph node metastasis, and integration of molecular and radiomic features for non-invasive stratification [4,5,6]. Intraoperatively, AI applications such as augmented reality (AR), real-time image-guided navigation, and robotic-assisted thoracic surgery (RATS) have demonstrated increased surgical precision, reduced operative times, and enhanced safety [7,8,9]. These technologies facilitate individualized anatomical visualization, simulation, and intraoperative assistance, supporting both experienced and trainee surgeons [8]. Postoperative management benefits from AI-driven predictive models and wearable monitoring devices, enabling early complication detection and improved patient follow-up [10,11]. Despite these advances, significant challenges remain. Algorithmic bias, data integration, interpretability, regulatory barriers, and ethical concerns regarding data security and clinical accountability must be addressed to ensure safe and effective implementation [12]. The evolving synergy between human expertise and AI underscores the need for robust multicenter validation, standardized frameworks, and transparent, explainable outputs. Future research directions include digital twin technology, federated learning, and explainable AI to further improve reliability and accessibility [1,7]. As AI continues to mature, its integration promises to revolutionize thoracic surgery, driving the next generation of precision, safety, and patient-centered care. The aim of this review is to evaluate and synthesize the current landscape of artificial intelligence (AI) applications across the thoracic surgical pathway, from preoperative decision-support to intraoperative guidance and emerging autonomous interventions.

## 2. AI in Lung Cancer Screening

AI is becoming an essential component of lung cancer screening, especially in the interpretation of low-dose CT (LDCT) scans. AI algorithms, especially those based on deep learning and convolutional neural networks, have demonstrated high sensitivity (up to 94.6%) and specificity (up to 93.6%) for lung nodule detection, often matching or surpassing radiologist performance, especially for small nodules [13,14]. AI can reduce inter-reader variability, improve consistency in nodule measurement, and assist in nodule characterization, potentially decreasing unnecessary work-up of benign nodules and expediting diagnosis of malignant ones [15]. AI also enables workflow enhancements, such as radiation dose reduction through advanced image reconstruction, and can support radiologists in regions with limited expertise [16]. Implementation scenarios vary: using AI as a prescreener (where radiologists only review AI-positive scans) can reduce workload and maintain sensitivity, while AI as an assistant or backup may increase recall rates but also false positives and interpretation time [17]. AI-driven risk stratification and integration with clinical data may allow for personalized screening intervals and improved resource utilization. Despite these advances, limitations persist. Most AI models lack robust external validation, and clinical effectiveness and cost-effectiveness data remain limited and uncertain [18]. There is a risk of increased surveillance for indeterminate findings, and challenges remain regarding model generalizability, interpretability, and integration into clinical workflows [19]. In summary, AI offers substantial promise for improving lung cancer screening accuracy, efficiency, and workflow, but further large-scale, prospective validation and standardization are required before widespread clinical adoption.

## 3. AI in Laboratory Tests for Lung Cancer

The evaluation of bodily fluids—most commonly blood—offers a minimally invasive strategy for detecting tumor-related biomarkers, and is widely applied across the continuum of cancer care, including screening, diagnosis, therapeutic decision-making, and surveillance. Several circulating analytes, such as autoantibodies, complement components, microRNAs, tumor-derived DNA, and serum proteins, have been investigated as potential candidates for lung cancer screening [20]. Although these markers are promising, their diagnostic performance remains modest, and they are therefore regarded as adjunctive tools rather than stand-alone screening modalities. Predictive models that merge multi-marker panels with CT-based information and artificial intelligence have markedly improved the precision and reliability of early lung cancer detection. For example, during testing, the use of Artificial Neural Networks (ANNs) in combination with serum protein signatures (including β2-microglobulin, CEA, gastrin, CA125, NSE, sIL-6R, and three metal ions—Cu^2+^/Zn^2+^, Ca^2+^, and Mg^2+^) achieved an accuracy of approximately 85%. When clinical data—such as symptoms, risk factors, smoking exposure, and household environmental characteristics—were added, the prediction rate increased to 87.3% [21]. Furthermore, integrating CT-image analysis through the Pulmonary Nodules Artificial Intelligence Diagnostic System (PNAIDS) with tumor marker data yielded the highest reported specificity (96.1%), while combining PNAIDS with circulating abnormal cells resulted in a specificity of 94.1% [22]. These results collectively highlight the potential value of incorporating such multimodal, AI-driven approaches into lung cancer screening pathways. Overall, the adoption of AI-enhanced strategies holds considerable promise for advancing early detection and improving clinical outcomes. Continued research, validation, and clinical integration will be essential to ensure that these technologies are safe, dependable, and widely implementable, supported by ongoing collaboration between medical and computational disciplines.

## 4. AI in Lung Cancer Diagnosis

The diagnosis of lung cancer is mainly based on CT scans and tissue biopsies, methods that may sometimes result in misdiagnoses or missed cases [23]. Therefore, improving the sensitivity and specificity of noninvasive biomarkers is essential. Diagnosis is further complicated by variables such as tumor location, pathological subtype, presence of metastasis, and associated complications [24]. Artificial intelligence models have emerged as a valuable approach in lung cancer diagnosis, enhancing accuracy, consistency, and efficiency [25].

### 4.1. Imaging

Diagnosis often involves using CT and PET-CT (positron emission tomography-computed tomography) of the chest to identify abnormal masses or lung tumors. With the increasing adoption of AI, the medical community has recognized its potential to enhance diagnostic imaging. Recently, a deep learning (DL) algorithm was developed to detect lung cancer via low-dose CT scans, achieving an impressive AUC of 94.4% [26]. Another study analyzed CT scans of 200 lung nodules, reporting an AUC of 0.72 [27]. Additionally, machine learning (ML) has been applied to FDG-PET imaging for lung cancer detection, achieving sensitivities of 95.9% and 91.5% and specificities of 98.1% and 94.2% for standard and ultralow-dose scans, respectively, suggesting that ML approaches can detect lung cancer even at minimal radiation exposure of 0.11 mSv [28]. In a study by Sun et al., 395 pure ground glass nodules from 385 patients were randomly divided into a training set (n = 277) and a validation set (n = 118). Using radiomic features, a nomogram incorporating the RAD score, margin, spiculation, and nodule size was developed. The combined radiographic–radiomics model (AUC 0.77; 95% CI, 0.69–0.86) outperformed the radiographic-only model (AUC 0.71; 95% CI, 0.62–0.81) in predicting invasiveness in the validation set [28,29].

To further assess radiomics, a Chinese retrospective study evaluated 100 patients with solitary sub-solid nodules confirmed pathologically as minimally invasive or invasive adenocarcinoma. An integrated model combining CT-based features such as nodule size, shape, margins, and radiomic signatures was constructed, showing strong discrimination in both the training set (AUC 0.943) and validation set (AUC 0.912) [30]. These findings indicate that integrating ML-derived features with CT-based assessments can enhance the accuracy of tumor classification and invasiveness prediction. In a separate study of 301 lung carcinoma CT images, a deep convolutional neural network (DCNN)—a key deep learning approach—was used to identify lung cancer. The DCNN consisted of three convolutional layers, three pooling layers, and two fully connected layers. Training was performed on the authors’ dataset using a graphics processing unit, with images cropped and resampled to 256 × 256 pixels and augmented through rotation, flipping, and filtering to reduce overfitting. Probabilities for three cancer types were estimated, and threefold cross-validation reported a sensitivity of 0.93, precision of 0.82, and overall accuracy of approximately 71%, comparable to that of cytotechnologists and pathologists. This DCNN model further distinguished small-cell lung carcinoma, adenocarcinoma, and squamous-cell lung carcinoma, with sensitivity, specificity, and F1 scores of 0.90, 0.44, and 0.59, respectively [31]. Finally, Saad et al. demonstrated that radiomics could differentiate NSCLC from peripherally located small-cell lung cancer (SCLC), achieving an AUC of 0.93 [32]. While pathological analysis remains the standard for revealing phenotypic differences through invasive methods such as biopsies or resections, AI-driven imaging has the potential to detect lung cancer subtypes noninvasively.

### 4.2. Histopathology

Histological assessment—typically obtained via bronchoscopy or percutaneous needle biopsy—remains the diagnostic gold standard for lung cancer. However, manual interpretation can be challenging because of the wide variety of pathological subtypes. In a study by Yu, a dataset of 2480 histopathological images from lung squamous cell carcinoma and adenocarcinoma was analyzed, and the algorithm was able to distinguish malignant tumors from normal tissues with an AUC of 0.81 [33]. Teramoto et al. evaluated 298 images using DCNNs and achieved classification accuracies of 89% for adenocarcinoma, 60% for squamous cell carcinoma, and 70% for small-cell lung cancer—outperforming both cytotechnologists and pathologists [31]. In another prospective investigation, a predictive model incorporating clinical parameters (age and smoking status), radiologic characteristics of pulmonary nodules (including nodule size, number, upper-lobe location, malignant edge features, and subsolid appearance), LDCT-derived AI outputs, and liquid biopsy data demonstrated optimal performance in the training cohort, with a sensitivity of 89.53%, specificity of 81.31%, and an AUC of 0.880 [34]. This integrated approach may enhance the early detection of lung cancer while reducing unnecessary surgical interventions in patients with benign findings. Taken together, these results suggest that AI-supported histopathologic evaluation has the potential to improve diagnostic accuracy and efficiency while reducing the likelihood of misclassification [35].

### 4.3. Biomarkers

Key biomarkers associated with lung cancer include Rb, K-RAS, EGFR, c-MET, TP53, ALK, and PD-L1 [36]. Although numerous candidates have been proposed, their practical use remains constrained by variability in diagnostic performance and prognostic value. AI-driven proteomic approaches are now being used to explore multi-marker panels to improve the detection of different lung cancer subtypes. Coudray et al. proposed that specific gene alterations might influence the morphological appearance of cancer cells on histologic slides; by training neural networks, they were able to predict the ten most frequent mutations in lung adenocarcinoma [36]. Six of these mutations (KRAS, STK11, TP53, EGFR, SETBP1, and FAT1) were identifiable from pathology images with accuracies ranging from 73.3% to 85.6% [36]. Additional studies have reported that integrating multiple biomarkers—such as human epididymis protein 4 (HE4), soluble vascular cell adhesion molecule-1 (sVCAM-1), transthyretin (TTR), apolipoprotein A2 (ApoA2), and the carcinoembryonic antigen CEA—can markedly enhance diagnostic performance. One such model reached a sensitivity of 93.33%, specificity of 92.00%, and an AUC of 0.988, indicating excellent discriminative ability [24]. Since no universally accepted biomarker panel exists for lung cancer, each proposed combination requires population-specific testing and validation before clinical implementation. Overall, these findings suggest that deep-learning-based analysis has the potential to support pathologists in identifying tumor subtypes and genetic mutations, and may ultimately assist clinicians in the early diagnosis and screening of lung cancer.

## 5. AI in Lung Cancer Staging

Lung cancer remains one of the most commonly diagnosed malignancies and continues to be the leading cause of cancer-related mortality worldwide [37]. Accurate staging is essential, as it guides treatment planning and helps predict patient outcomes. Non-small-cell lung cancer is classified from stage I to IV based on clinical, radiological, and pathological findings, whereas small-cell lung cancer is typically categorized into limited or extensive disease. The TNM system (tumor, node, metastasis) is the standard framework for staging. Timely staging generally relies on imaging modalities such as CT and PET. Unfortunately, many lung cancers are identified at advanced stages, contributing to poor survival rates [38]. AI technologies are well suited to managing large volumes of repetitive, image-based tasks, making them valuable tools for assisting clinicians in visually intensive workflows. By expediting interpretation of CT scans and pathology slides, AI has the potential to streamline and enhance the accuracy of lung cancer staging. When applied as a second reader for CT and PET imaging, AI can reduce radiologists’ workload while improving the detection of suspicious nodules. PET imaging is particularly useful for assessing metastatic spread, whereas CT provides information on local tumor invasion [39]. However, staging accuracy can be influenced by the radiologist’s experience, and the complex appearance of lung nodules on CT increases the risk of interpretive errors. Manual review may therefore lead to missed or incorrect diagnoses, complicating efforts to identify lung cancer early [40]. Minimizing observational variability is essential, and AI represents a promising solution for reducing such errors and supporting more consistent staging practices [41].

## 6. Ai in Lung Cancer Treatment

Recent developments highlight the growing role of AI as an important tool in lung cancer management. Techniques such as DL and radiomics are increasingly contributing to clinical decision-making by offering quantitative analyses of patient data. These models are well suited to handle the complexity, variability, and biological heterogeneity that characterize lung cancer. Treatment options for lung cancer include both surgical and non-surgical approaches, such as radiotherapy, chemotherapy, and immunotherapy. Radiotherapy remains a cornerstone of treatment, and AI has demonstrated significant potential in improving its planning and delivery. Machine learning systems can integrate the substantial amount of high-quality data produced during radiotherapy—CT imaging, dose distributions, and treatment records—to refine treatment planning. ML methods have been used to optimize beam configurations, estimate dose–volume histograms, evaluate radiation dose and toxicity, and support clinical decision-making [42]. Together, these predictive tools may enable more individualized radiotherapy with enhanced safety and precision. Immunotherapy still poses challenges regarding patient selection and predicting therapeutic benefit. ML and radiomic analyses, however, offer non-invasive means to better evaluate the tumor and its microenvironment, thereby aiding in anticipating treatment responses. ML approaches have been applied to identify biological indicators of tumor immunogenicity and to design scoring systems that predict outcomes following immune checkpoint inhibitor (CPI) therapy. Radiomic signatures derived from CT scans have similarly been explored as predictors of immunotherapy response [42]. Kureshi et al. proposed a data-driven predictive model for assessing tumor response to EGFR-TKI treatment in advanced NSCLC; by incorporating clinical profiles, environmental exposures, and EGFR mutation status, the model reached an accuracy of 76% [42]. Beyond guiding therapeutic decisions, AI holds promise in accelerating drug discovery—both by identifying new applications for existing drugs and by pinpointing candidates for future trials. Neural network (NN) models have also been used to forecast postoperative outcomes in NSCLC, demonstrating strong performance in predicting cardio-respiratory toxicity and postoperative complications. Such results underscore the expanding role of AI in drug development and in assessing patient risk profiles [43].

Digital twin technology in oncologic thoracic surgery refers to the creation of a dynamic, virtual replica of an individual patient that integrates multimodal clinical data—including imaging, genomics, pathology, and real-time physiologic parameters—using artificial intelligence and advanced computational modeling. This digital representation enables simulation of disease progression, prediction of treatment response, and optimization of personalized therapeutic strategies in lung cancer and other thoracic malignancies [44]. Federated learning is a collaborative artificial intelligence approach that enables multiple institutions to train shared machine learning models on local data without transferring patient-level information, thereby preserving privacy and data security. In oncologic thoracic surgery, federated learning allows for the development of robust AI models for lung cancer diagnosis, risk stratification, and surgical decision support by leveraging diverse, multi-institutional datasets while maintaining compliance with data protection regulations [44].

### 6.1. Surgical Eligibility

Determining surgical eligibility during preoperative planning is often a multifaceted decision, encompassing scientific, ethical, and legal considerations, particularly in patients with pre-existing respiratory or cardiovascular conditions. Traditional risk assessment tools, such as the Goldman index for cardiac risk and the Torrington index for respiratory risk, are effective in categorizing patients into broad risk categories but fall short in providing precise, individualized predictions of operative risk [44]. Esteva H, et al. investigated the use of artificial NN—modeled after the human neural system—for estimating postoperative outcomes following lung resection, comparing them to conventional risk indices. NN demonstrated greater flexibility and individualized predictive power, achieving nearly 100% sensitivity and specificity for patient outcomes [44]. Similarly, Santos-Garcia G, et al. reported that artificial NN models exhibited high accuracy in forecasting postoperative cardiorespiratory complications [45]. In lung cancer surgery, conventional approaches like video-assisted thoracic surgery (VATS) have shown advantages, including reduced surgical trauma, faster recovery, and lower complication rates. Nevertheless, these techniques have limitations, such as restricted visualization and limited instrument maneuverability. AI has the potential to transform surgical practice by offering real-time analysis during operations, supporting enhanced decision-making, and improving overall surgical outcomes. Chang et al. explored the role of AI in pre-anesthetic consultations, highlighting the increasing trend toward comprehensive digitalization in healthcare. Their study demonstrates AI’s capability to leverage historical medical data for accurate, noninvasive predictions. The AI-assisted predictive model not only enables integrated risk assessments but also accommodates dynamic adjustments through clinician input, allowing it to adapt to a wide range of patient records [46]. Etienne et al. [5] reported successful applications of AI in predicting cardiorespiratory complications and postoperative prognosis in non-small-cell lung cancer patients. Their findings support Chang et al.’s observation that AI, particularly the Naïve Bayes Classifier, is highly effective for predictive modeling. Both studies emphasize the potential of AI to enhance collaboration among healthcare professionals, including applications such as differentiating lung adenocarcinoma from squamous cell carcinoma and surpassing pulmonologists in interpreting pulmonary function tests [5,46].

Despite these advances, both studies acknowledge barriers to the widespread adoption of AI. They note the limitations of traditional equation-based approaches, contrasting them with AI’s adaptability and real-time processing capabilities. Additionally, they stress the importance of AI in addressing complex clinical scenarios, particularly in patients with multiple comorbidities, to achieve full integration into routine clinical practice [5,46].

### 6.2. Intraoperative Support

Kanavati et al. emphasize how deep-learning (DL) systems can provide critical support during surgery by offering real-time guidance. Trained on large, high-quality datasets, these DL models are able to delineate tumor margins intraoperatively with a level of accuracy that exceeds human visual assessment. Such precision helps reduce the likelihood of accidental injury to surrounding tissues and supports a more complete and safer tumor resection. Their work illustrates how integrating AI with intraoperative imaging tools can enhance surgeons’ situational awareness and improve decision-making in the operating room’s rapidly changing environment [47]. Intraoperative imaging has progressed considerably, moving beyond conventional X-rays to include C-arm systems, intraoperative ultrasound, and intraoperative MRI. Molecular imaging has also expanded the field, especially through radio-guided surgery using radiotracers, fluorescent probes, magnetic agents, or hybrid combinations. Novel modalities—including multispectral optoacoustic tomography (MSOT), fiber-based microscopy, and Raman spectroscopy—further enrich the available imaging toolkit. Approaches traditionally used for preoperative navigation are now being applied seamlessly to intraoperative molecular imaging. Technologies such as freehand SPECT, enhanced with augmented-reality overlays and pointer-based navigation, demonstrate how these systems can be combined. Their ability to adapt to tissue deformation and to provide immediate confirmation of target localization through radio- or fluorescence-based feedback highlights their value in guiding complex procedures [48]. Li et al. introduce an innovative strategy that merges three-dimensional (3D) printing with augmented-reality (AR) visualization. By producing patient-specific 3D-printed lung models, surgeons can better understand anatomical relationships before entering the operating room, overcoming the limitations of two-dimensional screen-based views. When these models are paired with AR during surgery, spatial orientation improves significantly, contributing to reduced operative duration, decreased blood loss, and shorter hospitalization [49]. Beyond their use in resection planning, 3D printing and AR have shown utility in tasks such as mapping intersegmental planes during segmentectomy, suggesting their broader transformative impact. These technologies also hold considerable educational value, offering trainees a tactile and immersive way to learn complex pulmonary anatomy. Addressing the challenges of planning pulmonary segmentectomies, the PulmoVR (Virtual Reality) platform provides an AI- and VR-based solution capable of rapidly converting patient CT scans into an interactive three-dimensional environment. Its advantages—speed, cost-efficiency, and intuitive immersive visualization—demonstrate the potential of virtual reality to deepen anatomical understanding and enhance surgical preparation.

AI-driven computer vision algorithms are used for real-time anatomical recognition and segmentation during robotic-assisted thoracic procedures. These systems can identify critical structures (e.g., vessels, bronchi, tumors) intraoperatively, enhancing surgical precision and reducing the risk of inadvertent injury. For example, AI-powered image analysis can overlay augmented reality guidance onto the surgeon’s console, facilitating safer dissection and resection. Automated skill assessment and workflow optimization are implemented via machine learning models that analyze instrument motion and surgical video data. These models provide feedback on technique, efficiency, and adherence to procedural steps, supporting both intraoperative decision-making and post-procedure training. This integration is particularly relevant for complex thoracic procedures, where AI can help standardize performance and accelerate the learning curve for less experienced surgeons. Robotic platforms are increasingly incorporating AI for semi-autonomous or autonomous task execution, such as suture placement, camera control, and instrument positioning. While current clinical use remains at the level of robot assistance (autonomy level 1), research is progressing toward higher autonomy, with conditional autonomy demonstrated in preclinical models [50]

Together, these studies illustrate a landscape in which AI, AR, and VR increasingly intersect to refine surgical workflows and elevate clinical performance [51].

### 6.3. Real-Time Decision Support

Liu et al. introduce an interactive human–machine interface (HMI) that incorporates a mobile optical coherence tomography (OCT) platform, deep learning techniques, and attention modules to address a key limitation of frozen-section analysis—namely, the difficulty in defining resection margins when tumor histology is not known before surgery. This HMI can highlight suspicious areas on live images and automatically assess tumor grade, thereby supporting intraoperative decision-making [52]. In their study, twelve patients with lung tumors of unknown preoperative histology—ultimately diagnosed as adenocarcinoma—underwent thoracoscopic resection, during which the AI-enhanced system was applied to evaluate freshly excised tissue. Its performance was compared with conventional frozen sections, using final paraffin-embedded pathology as the reference standard. The AI approach demonstrated markedly superior discrimination among minimally invasive adenocarcinoma (MIA), invasive adenocarcinoma (IA), and normal lung tissue, achieving an overall accuracy of 84.9%, whereas frozen sections reached only 20%. Sensitivity and specificity were also higher for both MIA (89% and 82.7%) and IA (94% and 80.6%), respectively. These findings indicate that the proposed system could offer faster and more reliable intraoperative diagnostic support, potentially improving surgical outcomes [52]. Similarly, the work of Pao et al. underscores the growing contribution of AI to intraoperative decision support. Their study highlights how deep learning models can detect fine-grained pathological patterns during surgery and deliver immediate feedback about tissue characteristics. Such real-time histologic assessment allows surgeons to adjust their operative strategy as needed, promoting complete tumor removal while avoiding unnecessary excision of healthy structures. In this way, integrating AI-based analytical tools directly into the operative workflow may refine intraoperative judgment by complementing surgical expertise with rapid computational insight [53].

### 6.4. Autonomy in Surgical Artificial Intelligence

Understanding autonomy in surgical AI requires a structured framework that distinguishes between simple assistance and full automation. The most widely adopted classification system defines five levels of autonomy in surgical robotics (LASR): Level 1 (Robot Assistance), where the surgeon maintains complete control with robotic instruments providing enhanced dexterity; Level 2 (Task Autonomy), where the system can execute specific subtasks under surgeon supervision; Level 3 (Conditional Autonomy), where the robot performs entire tasks autonomously but requires surgeon oversight and intervention capability; Level 4 (High Autonomy), where the system operates independently for complete procedures with minimal human intervention; and Level 5 (Full Autonomy), where the robot performs surgery entirely without human involvement. Current surgical robots in thoracic surgery operate predominantly at Level 1 (Robot Assistance), with 86% of FDA-cleared surgical robots functioning at this level. The da Vinci surgical system, the dominant platform in thoracic surgery, remains a telemanipulation device where the surgeon controls every movement through a master console. A small proportion of systems (6%) have achieved Level 3 (Conditional Autonomy), capable of executing specific tasks such as suturing or tissue manipulation autonomously under surgeon supervision. Critically, no clinical investigations of autonomous surgical AI on human patients have been reported, with all studies remaining at preclinical stages using in silico simulations, ex vivo models, or animal experiments [54].

### 6.5. Postoperative Support

Following lung cancer surgery, the postoperative phase represents a crucial period in which AI-driven tools are increasingly demonstrating value in pathological evaluation. Several investigations underline how deep learning techniques can assist in detailed histologic classification [55,56]. These studies addressed a complex five-category classification task—distinguishing lepidic, acinar, papillary, micropapillary, and solid growth patterns—and showed that AI can support both accurate subtype identification and a deeper characterization of tumor morphology. Given the well-known heterogeneity of lung cancer and the interpretative challenges it presents, enhanced precision in postoperative histologic subtyping is particularly important. In addition, postoperative management requires careful assessment of surgical margins, another area where AI-based tools may offer assistance.

Turning to prognosis, AI has also emerged as a key contributor in modeling the diverse variables that affect patient outcomes, such as age, tumor biology, and therapeutic strategies. Within this context, predictive systems like the ITEN model (impact of treatment evolution in non-small-cell lung cancer) provide tailored recommendations for systemic therapies, for example in patients presenting with bone metastases, with the aim of improving survival. The development of AI-enhanced prognostic models—especially those integrating neural networks—marks a significant advancement in optimizing clinical decision-making. The ITEN model’s alignment with previously published evidence further supports its robustness in forecasting survival in individuals with non-small-cell lung cancer [57]. In Table 1 and Table 2, we summarize key clinical applications of artificial intelligence, the main artificial intelligence methodologies and their applications in thoracic surgery [Table 1 and Table 2].

## 7. Limitations and Future Perspectives

A key issue emerging from this review is the persistent problem of limited interpretability in several AI applications. Although many models demonstrate strong performance in classification tasks, little progress has been made in improving common-sense reasoning—particularly when it comes to understanding subtle cellular or tissue-level characteristics. This lack of explainability creates obstacles in clinical practice, where physicians rely on transparent reasoning to guide treatment decisions. When AI outputs appear opaque or difficult to interpret, it may undermine clinician confidence and slow down broader clinical adoption. Many of the reviewed studies also highlight the data-intensive nature of modern deep learning systems. These algorithms typically require very large volumes of high-quality, labeled data. In practice, however, generating extensive annotations depends heavily on expert pathologists, making the process slow and resource-intensive. The problem becomes even more pronounced when dealing with rare tumor subtypes or unusual molecular signatures, where only limited data exist. Consequently, the lack of sufficiently diverse datasets restricts the generalizability of AI models.

Although deep learning excels in image-based classification, the reviewed evidence shows that it performs less effectively on more complex analytical tasks—such as regression, clustering, or the integration of multidimensional clinical variables. In these contexts, conventional machine learning methods may still outperform deep learning. This underscores the need to match the choice of AI technique with the specific analytical requirements of each clinical application. Another recurring challenge involves the limited ability of deep learning models to generalize across institutions or imaging conditions. Overfitting—where a model learns patterns specific to the training dataset but struggles with new or heterogeneous data—remains a significant barrier. Given the variability in imaging protocols, scanner types, and patient populations in lung cancer care, achieving reliable generalization is difficult. The computational burden of deep learning also poses practical constraints. As several studies point out, high-resolution biopsy images require substantial processing power and memory. This raises concerns about the feasibility of deploying such models in routine clinical settings, particularly in resource-limited environments. While ongoing research is focused on improving computational efficiency, the need for powerful hardware continues to be a limiting factor.

The integration of AI into surgical workflows also brings ethical considerations to the forefront. Issues related to data privacy, algorithmic transparency, and potential biases must be addressed to ensure safe and equitable use. Crucially, AI should complement—rather than replace—the human elements of care, such as empathy, clinical judgement, and nuanced decision-making. Protecting patient autonomy, ensuring informed consent, and minimizing the risk of algorithm-driven disparities are essential as AI becomes more embedded in clinical practice. Looking ahead, constructive collaboration among clinicians, ethicists, data scientists, policymakers, and—most importantly—patients will be crucial. A shared commitment to transparency, fairness, and open communication can ensure that the rapid evolution of AI aligns with the fundamental values of medicine. By doing so, technological innovation can strengthen, rather than compromise, human dignity, autonomy, and the trust that forms the foundation of patient care [58]. Artificial intelligence in thoracic surgery demonstrates potential for cost-effectiveness primarily by improving diagnostic accuracy, reducing unnecessary procedures, and optimizing resource utilization. In lung cancer screening, AI-assisted CT interpretation has been shown to lower costs and increase effectiveness compared to standard radiologist-only workflows, with robust modeling indicating a negative incremental cost-effectiveness ratio (ICER) and sustained value across sensitivity analyses. These economic benefits are driven by earlier detection, fewer missed diagnoses, and streamlined care pathways. AI integration into surgical planning and intraoperative guidance can reduce operative times and complication rates, which translates to lower direct healthcare costs and improved quality-adjusted life years (QALYs). However, the initial investment in AI infrastructure—including software, hardware, and staff training—remains substantial, and the economic impact is sensitive to implementation costs and local resource constraints. Dynamic modeling suggests that long-term value is maximized when adaptive learning and workflow integration are considered, but indirect costs and equity issues are often underreported in current analyses [59]. In Table 3, we summarized the most significative Artificial Intelligence Studies in Surgery [Table 3].

## 8. Conclusions

AI is rapidly transforming oncologic thoracic surgery, advancing from decision support in diagnostics and surgical planning to integration with robotic-assisted and image-guided interventions. AI-driven tools now enhance tumor detection, risk stratification, intraoperative navigation, and postoperative monitoring, improving precision and workflow efficiency. However, widespread adoption requires rigorous multicenter validation, standardized frameworks, and robust ethical governance to address challenges such as algorithmic bias, data security, and clinical accountability.

Clinicians should actively participate in multidisciplinary AI development teams to ensure that models address clinically relevant questions and integrate seamlessly into existing workflows. Engagement with AI vendors and developers is essential to advocate for transparency in algorithmic design, data provenance, and model limitations.

Surgeons must maintain vigilance regarding ethical considerations, including patient autonomy, informed consent for AI-assisted decision-making, data privacy, and accountability for AI-generated recommendations. The preservation of bedside clinical assessment and human intuition remains paramount, with AI serving as an augmentative tool rather than a replacement for surgical expertise.

Researchers must prioritize rigorous validation methodologies to establish clinical utility and safety of AI applications in thoracic surgery. Future studies should emphasize multicenter external validation, prospective real-time validation in clinical settings, and demonstration of improved patient outcomes rather than solely algorithmic performance metrics. With responsible implementation, AI will play a pivotal role in the evolution toward precision and potentially autonomous thoracic oncologic surgery.

## Figures and Tables

**Table 1:** Key clinical applications of artificial intelligence in thoracic surgery.

Application Area	Clinical Utility/Examples
Diagnostic Imaging & Radiomics	Automated detection/classification of lung nodules, cancer staging, lymph node analysis
Preoperative Planning	3D reconstruction, anatomical segmentation, risk stratification, surgical simulation
Intraoperative Guidance	Real-time image-guided navigation, augmented reality, robotic-assisted surgery
Postoperative Management	Predictive modeling for complications, wearable monitoring, outcome prediction
Pathology & Biomarker Analysis	AI-based histological classification, molecular subtyping, response prediction
Workflow & Resource Optimization	Operating room scheduling, case prioritization, surgical skill assessment
Education & Training	AI-assisted surgical video analysis, simulation, quality evaluation

**Table 2:** Overview of artificial intelligence methodologies and their applications in thoracic surgery.

Type of AI	Definition/Methodology	Key Applications in Thoracic Surgery	Advantages	Limitations/Challenges
Machine Learning (ML)	Algorithms that learn patterns from structured data (e.g., logistic regression, random forests, support vector machines).	Preoperative risk assessment, outcome prediction, diagnostic support, perioperative planning.	Handles diverse data types; interpretable models; useful for structured clinical data.	Requires high-quality labeled data; limited in handling complex imaging data; risk of bias.
Deep Learning (DL)	Subset of ML using multi-layer neural networks (e.g., convolutional neural networks) for complex data, especially images.	Automated image interpretation (CT, X-ray), nodule detection, intraoperative guidance, skill assessment.	Superior performance in image analysis; can extract subtle features; scalable to large datasets.	Requires large annotated datasets; less interpretable (“black box”); high computational cost.
Radiomics	Extraction of quantitative features from medical images, often combined with ML/DL for predictive modeling.	Lung nodule characterization, tumor staging, histology prediction, outcome prediction, personalized treatment planning.	Non-invasive; captures tumor heterogeneity; enhances diagnostic and prognostic accuracy.	Standardization issues; external validation needed; sensitive to imaging protocols.

**Table 3:** Artificial Intelligence Studies in Surgery.

Study (First Author, Year)	Prospective or Retrospective	Number of Patients	Validation (Internal/External/Other Hospital)	Proximity to OR Use	References
Loftus TJ, 2023	Retrospective (scoping review of 36 studies)	163–2,882,526 (per study)	Mostly internal (80.6%), some external (13.8%), few real-time (5.6%)	Most tools are not in clinical use; 36.1% present implementation frameworks, none tested in OR	[60]
Kenig N, 2024	Systematic review (102 studies)	2,837,211 (aggregate)	45% high-evidence validation, 14% public datasets; mix of internal/external	Mainly preoperative risk assessment; few models close to OR use	[61]
Vasey B, 2023	Scoping review (129 studies)	Not specified (preclinical, animal, ex vivo, in silico)	All studies at preclinical stage; no human clinical validation	All tools at development stage; none in human OR use	[50]
Paracchini S, 2025	Systematic review (21 studies)	Not specified	Internal validation predominant; external validation rare	Video analysis tools for phase/instrument recognition; not yet routine in OR	[62]
Stam WT, 2022	Systematic review (15 studies)	Not specified	Both internal and external validation reported	AI for complication prediction; not yet routine in OR	[63]
Bellini V, 2024	Systematic review (22 studies)	Not specified	Internal validation common; external validation limited	AI for OR management; some tools piloted, not routine	[64]
Limon D, 2025	Narrative review	Not specified	Not specified	Highlights emerging models; most not yet in routine OR use	[65]

## Data Availability

Data are available on request.

## Abbreviations

AI	Artificial intelligence
ANN	Artificial neural network
AR	Augmented reality
CPI	Checkpoint inhibitor
CT	Computed tomography
DCNN	Deep convolutional neural network
DL	Deep learning
DLA	Deep learning algorithm
EGFR	Epidermal growth factor
HMI	Human–machine interface
IA	Invasive adenocarcinoma
ITEN	Impact of treatment evolution in non-small-cell lung cancer
LDCT	Low-dose CT
MIA	Minimally invasive adenocarcinoma
ML	Machine learning
MSOT	Multispectral optoacoustic tomography
NN	Neural network
NSCLC	Non-small-cell lung cancer
OCT	Optical coherence tomography
PET-CT	Positron emission tomography–computed tomography
PNAIDS	Pulmonary Nodules Artificial Intelligence Diagnostic System
RATS	Robotic-assisted thoracic surgery
SCLC	Small-cell lung cancer
TKI	Tyrosine kinase inhibitor
VR	Virtual reality

## References

[1] Leivaditis V., Maniatopoulos A.A., Lausberg H., Mulita F., Papatriantafyllou A., Liolis E., Beltsios E., Adamou A., Kontodimopoulos N., Dahm M. (2025). Artificial intelligence in thoracic surgery: A review bridging innovation and clinical practice for the next generation of surgical care. J. Clin. Med..

[2] Bellini V., Valente M., Del Rio P., Bignami E. (2021). Artificial intelligence in thoracic surgery: A narrative review. J. Thorac. Dis..

[3] Abbaker N., Minervini F., Guttadauro A., Solli P., Cioffi U., Scarci M. (2024). The future of artificial intelligence in thoracic surgery for non-small cell lung cancer treatment: A narrative review. Front. Oncol..

[4] Mank Q.J., Thabit A., Maat A.P.W.M., Siregar S., Mahtab E.A.F., van Walsum T., Sadeghi A.H., Kluin J. (2025). State-of-the-art artificial intelligence methods for pre-operative planning of cardiothoracic surgery and interventions: A narrative review. J. Thorac. Dis..

[5] Etienne H., Hamdi S., Le Roux M., Camuset J., Khalife-Hocquemiller T., Giol M., Debrosse D., Assouad J. (2020). Artificial intelligence in thoracic surgery: Past, present, perspective and limits. Eur. Respir. Rev..

[6] Rizzo S.M., Kalra M.K., Schmidt B., Raupach R., Maher M.M., Blake M.A., Saini S. (2005). CT images of abdomen and pelvis: Effect of nonlinear three-dimensional optimized reconstruction algorithm on image quality and lesion characteristics. Radiology.

[7] Roche J.J., Seyedshahi F., Rakovic K., Thu A.W., Le Quesne J., Blyth K.G. (2025). Current and future applications of artificial intelligence in lung cancer and mesothelioma. Thorax.

[8] Cusumano G., D’Arrigo S., Terminella A., Lococo F. (2024). Artificial intelligence applications for thoracic surgeons: “The phenomenal cosmic powers of the magic lamp”. J. Clin. Med..

[9] Chen Z., Zhang Y., Yan Z., Dong J., Cai W., Ma Y., Jiang J., Dai K., Liang H., He J. (2021). Artificial intelligence-assisted display in thoracic surgery: Development and possibilities. J. Thorac. Dis..

[10] Kim Y., Park J.Y., Hwang E.J., Lee S.M., Park C.M. (2021). Applications of artificial intelligence in the thorax: A narrative review focusing on thoracic radiology. J. Thorac. Dis..

[11] Yang D., Miao Y., Liu C., Zhang X., Wang L., Li J., Zhao H., Chen Q., Zhou Y., Sun K. (2024). Advances in artificial intelligence applications in the field of lung cancer. Front. Oncol..

[12] Platz J.J., Bryan D.S., Ferguson M.K., Naunheim K.S. (2025). Surgeon perception of artificial intelligence in thoracic surgery: Insights from an international survey. Ann. Thorac. Surg..

[13] Thong L.T., Chou H.S., Chew H.S.J., Lau Y. (2023). Diagnostic test accuracy of artificial intelligence-based imaging for lung cancer screening: A systematic review and meta-analysis. Lung Cancer.

[14] Adams S.J., Stone E., Baldwin D.R., Callister M.E.J., Hansell D.M., McRonald F., Parmar A., Screaton N.J., Waller D.A., Woolhouse I. (2023). Lung cancer screening. Lancet.

[15] Wu Q., Huang Y., Wang S., Qi L., Zhang Z., Hou D., Li H., Zhao S. (2024). Artificial intelligence in lung cancer screening: Detection, classification, prediction, and prognosis. Cancer Med..

[16] Cellina M., Cacioppa L.M., Cè M., Chiarpenello V., Costa M., Vincenzo Z., Pais D., Bausano M.V., Rossini N., Bruno A. (2023). Artificial intelligence in lung cancer screening: The future is now. Cancers.

[17] Lee M., Hwang E.J., Lee J.H., Nam J.G., Lim W.H., Park H., Park C.M., Choi H., Park J., Goo J.M. (2025). Artificial intelligence for low-dose CT lung cancer screening: Comparison of utilization scenarios. Am. J. Roentgenol..

[18] Geppert J., Auguste P., Asgharzadeh A., Ghiasvand H., Patel M., Brown A., Jayakody S., Helm E., Todkill D., Madan J. (2025). Software with artificial intelligence-derived algorithms for detecting and analysing lung nodules in CT scans: Systematic review and economic evaluation. Health Technol. Assess..

[19] Ayasa Y., Alajrami D., Idkedek M., Tahayneh K., Akar F.A. (2025). The impact of artificial intelligence on lung cancer diagnosis and personalized treatment. Int. J. Mol. Sci..

[20] Casillas C.E.H., Fernández J.M.F., Camberos E.P., López E.J.H., Pacheco G.L., Velázquez M.M. (2014). Current status of circulating protein biomarkers to aid the early detection of lung cancer. Future Oncol..

[21] Yang H., Chen H., Zhang G., Li H., Ni R., Yu Y., Zhang Y., Wu Y., Liu H. (2022). Diagnostic value of circulating genetically abnormal cells to support computed tomography for benign and malignant pulmonary nodules. BMC Cancer.

[22] Liu M., Wu J., Wang N., Zhang X., Bai Y., Guo J., Zhang L., Liu S., Tao K. (2023). The value of artificial intelligence in the diagnosis of lung cancer: A systematic review and meta-analysis. PLoS ONE.

[23] Pei Q., Luo Y., Chen Y., Li J., Xie D., Ye T. (2022). Artificial intelligence in clinical applications for lung cancer: Diagnosis, treatment and prognosis. Clin. Chem. Lab. Med..

[24] Huang S., Yang J., Shen N., Xu Q., Zhao Q. (2023). Artificial intelligence in lung cancer diagnosis and prognosis: Current application and future perspective. Semin. Cancer Biol..

[25] Delzell D.A.P., Magnuson S., Peter T., Smith M., Smith B.J. (2019). Machine learning and feature selection methods for disease classification with application to lung cancer screening image data. Front. Oncol..

[26] Ardila D., Kiraly A.P., Bharadwaj S., Choi B., Reicher J.J., Peng L., Tse D., Etemadi M., Ye W., Corrado G. (2019). End-to-end lung cancer screening with three-dimensional deep learning on low-dose chest computed tomography. Nat. Med..

[27] Schwyzer M., Ferraro D.A., Muehlematter U.J., Curioni-Fontecedro A., Huellner M.W., von Schulthess G.K., Kaufmann P.A., Burger I.A., Messerli M. (2018). Automated detection of lung cancer at ultralow dose PET/CT by deep neural networks: Initial results. Lung Cancer.

[28] Sun Y., Li C., Jin L., Gao P., Zhao W., Ma W., Tan M., Wu W., Duan S., Shan Y. (2020). Radiomics for lung adenocarcinoma manifesting as pure ground-glass nodules: Invasive prediction. Eur. Radiol..

[29] Feng B., Chen X., Chen Y., Li Z., Hao Y., Zhang C., Li R., Liao Y., Zhang X., Huang Y. (2019). Differentiating minimally invasive and invasive adenocarcinomas in patients with solitary sub-solid pulmonary nodules with a radiomics nomogram. Clin. Radiol..

[30] Chen B.T., Chen Z., Ye N., Mambetsariev I., Fricke J., Daniel E., Wang G., Wong C.W., Rockne R.C., Colen R.R. (2020). Differentiating peripherally located small-cell lung cancer from non-small cell lung cancer using a CT radiomic approach. Front. Oncol..

[31] Teramoto A., Tsukamoto T., Kiriyama Y., Fujita H. (2017). Automated classification of lung cancer types from cytological images using deep convolutional neural networks. BioMed Res. Int..

[32] Saad M., Choi T.S. (2018). Computer-assisted subtyping and prognosis for non-small cell lung cancer patients with unresectable tumor. Comput. Med. Imaging Graph..

[33] Yu K.H., Zhang C., Berry G.J., Altman R.B., Ré C., Rubin D.L., Snyder M. (2016). Predicting non-small cell lung cancer prognosis by fully automated microscopic pathology image features. Nat. Commun..

[34] Scott A., Salgia R. (2008). Biomarkers in lung cancer: From early detection to novel therapeutics and decision making. Biomark. Med..

[35] Genovese E., Canì A., Rizzo S., Angeretti M.G., Leonardi A., Fugazzola C. (2011). Comparison between MRI with spin-echo echo-planar diffusion-weighted sequence (DWI) and histology in the diagnosis of soft-tissue tumours. La Radiol. Medica.

[36] Coudray N., Ocampo P.S., Sakellaropoulos T., Narula N., Snuderl M., Fenyö D., Moreira A.L., Razavian N., Tsirigos A. (2018). Classification and mutation prediction from non-small cell lung cancer histopathology images using deep learning. Nat. Med..

[37] Rizzo S., Raimondi S., de Jong E.E.C., van Elmpt W., De Piano F., Petrella F., Bagnardi V., Jochems A., Bellomi M., Dingemans A.M. (2019). Genomics of non-small cell lung cancer (NSCLC): Association between CT-based imaging features and EGFR and KRAS mutations in 122 patients—An external validation. Eur. J. Radiol..

[38] Argentieri G., Valsecchi C., Petrella F., Jungblut L., Frauenfelder T., Del Grande F., Rizzo S. (2025). Implementation of the 9th TNM for lung cancer: Practical insights for radiologists. Eur. Radiol..

[39] Masood A., Sheng B., Li P., Hou X., Wei X., Qin J., Feng D. (2018). Computer-assisted decision support system in pulmonary cancer detection and stage classification on CT images. J. Biomed. Inform..

[40] Baker S.R., Patel R.H., Yang L., Lelkes V.M., Castro A. (2013). Malpractice suits in chest radiology: An evaluation of the histories of 8265 radiologists. J. Thorac. Imaging.

[41] Zhao W., Yang J., Sun Y., Li C., Wu W., Jin L., Yang Z., Ni B., Gao P., Wang P. (2018). 3D deep learning from CT scans predicts tumor invasiveness of subcentimeter pulmonary adenocarcinomas. Cancer Res..

[42] Kureshi N., Abidi S.S., Blouin C. (2016). A predictive model for personalized therapeutic interventions in non-small cell lung cancer. IEEE J. Biomed. Health Inform..

[43] Dercle L., Fronheiser M., Lu L., Du S., Hayes W., Leung D.K., Roy A., Wilkerson J., Guo P., Fojo A.T. (2020). Identification of non-small cell lung cancer sensitive to systemic cancer therapies using radiomics. Clin. Cancer Res..

[44] Esteva H., Marchevsky A., Nunez T., Luna C., Esteva M. (2002). Neural networks as a prognostic tool of surgical risk in lung resections. Ann. Thorac. Surg..

[45] Santos-García G., Varela G., Novoa N., Jiménez M.F. (2004). Prediction of postoperative morbidity after lung resection using an artificial neural network ensemble. Artif. Intell. Med..

[46] Chang Y., Hung K., Wang L., Yu C.-H., Chen C.-K., Tay H.-T., Wang J.-J., Liu C.-F. (2021). A real-time artificial intelligence-assisted system to predict weaning from ventilator immediately after lung resection surgery. Int. J. Environ. Res. Public Health.

[47] Kanavati F., Toyokawa G., Momosaki S., Rambeau M., Kozuma Y., Shoji F., Yamazaki K., Takeo S., Iizuka O., Tsuneki M. (2020). Weakly supervised learning for lung carcinoma classification using deep learning. Sci. Rep..

[48] Petrella F., Rizzo S.M.R., Rampinelli C., Casiraghi M., Bagnardi V., Frassoni S., Pozzi S., Pappalardo O., Pravettoni G., Spaggiari L. (2024). Assessment of pulmonary vascular anatomy: Comparing augmented reality by holograms versus standard CT images. Eur. Radiol. Exp..

[49] Li C., Zheng B., Yu Q., Yang B., Liang C., Liu Y. (2021). Augmented reality and 3-dimensional printing technologies for guiding complex thoracoscopic surgery. Ann. Thorac. Surg..

[50] Vasey B., Lippert K.A.N., Khan D.Z., Ibrahim M., Koh C.H., Horsfall H.L., Lee K.S., Williams S., Marcus H.J., McCulloch P. (2023). Intraoperative Applications of Artificial Intelligence in Robotic Surgery: A Scoping Review of Current Development Stages and Levels of Autonomy. Ann. Surg..

[51] Sadeghi A.H., Maat A.P.W.M., Taverne Y.J.H.J., Cornelissen R., Dingemans A.-M.C., Bogers A.J.C., Mahtab E.A. (2021). Virtual reality and artificial intelligence for 3-dimensional planning of lung segmentectomies. JTCVS Tech..

[52] Liu H.C., Lin M.H., Chang W.C., Zeng R.-C., Wang Y.-M., Sun C.-W. (2023). Rapid on-site AI-assisted grading for lung surgery based on optical coherence tomography. Cancers.

[53] Pao J.J., Biggs M., Duncan D., Lin D.I., Davis R., Huang R.S.P., Ferguson D., Janovitz T., Hiemenz M.C., Eddy N.R. (2023). Predicting EGFR mutational status from pathology images using a real-world dataset. Sci. Rep..

[54] Lee A., Baker T.S., Bederson J.B., Rapoport B.I. (2024). Levels of autonomy in FDA-cleared surgical robots: A systematic review. NPJ Digit. Med..

[55] Sheikh T.S., Kim J., Shim J., Cho M. (2022). Unsupervised learning based on multiple descriptors for WSIs diagnosis. Diagnostics.

[56] DiPalma J., Suriawinata A.A., Tafe L.J., Torresani L., Hassanpour S. (2021). Resolution-based distillation for efficient histology image classification. Artif. Intell. Med..

[57] Huang Z., Hu C., Chi C., Jiang Z., Tong Y., Zhao C. (2020). An artificial intelligence model for predicting 1-year survival of bone metastases in non-small-cell lung cancer patients based on XGBoost algorithm. BioMed Res. Int..

[58] Rad A.A., Vardanyan R., Athanasiou T., Maessen J., Nia P.S. (2025). The ethical considerations of integrating artificial intelligence into surgery: A review. Interdiscip. Cardiovasc. Thorac. Surg..

[59] El Arab R.A., Al Moosa O.A. (2025). Systematic review of cost effectiveness and budget impact of artificial intelligence in healthcare. NPJ Digit. Med..

[60] Loftus T.J., Altieri M.S., Balch J.A., Abbott K.L., Choi J., Marwaha J.S., Hashimoto D.A., Brat G.A., Raftopoulos Y., Evans H.L. (2023). Artificial Intelligence-enabled Decision Support in Surgery: State-of-the-art and Future Directions. Ann. Surg..

[61] Kenig N., Echeverria J.M., Vives A.M. (2024). Artificial Intelligence in Surgery: A Systematic Review of Use and Validation. J. Clin. Med..

[62] Paracchini S., Taliento C., Pellecchia G., Tius V., Tavares M., Borghi C., Buda A.A., Bartoli A., Bourdel N., Vizzielli G. (2025). Artificial intelligence in the operating room: A systematic review of AI models for surgical phase, instruments and anatomical structure identification. Acta Obstet. Gynecol. Scand..

[63] Stam W.T., Goedknegt L.K., Ingwersen E.W., Schoonmade L.J., Bruns E.R.J., Daams F. (2022). The prediction of surgical complications using artificial intelligence in patients undergoing major abdominal surgery: A systematic review. Surgery.

[64] Bellini V., Russo M., Domenichetti T., Panizzi M., Allai S., Bignami E.G. (2024). Artificial Intelligence in Operating Room Management. J. Med. Syst..

[65] Limon D., Satish V., Raghavan N., Nguyen P., Rajesh A. (2025). Artificial Intelligence in Surgery Revisited: A 2025 Guide to Understanding and Applying AI Models in Clinical Practice. Am. Surg..

